# Evaluation of risk factors for postpartum cerebral venous sinus thrombosis, a multicenter retrospective observational study

**DOI:** 10.1097/MD.0000000000040772

**Published:** 2024-12-06

**Authors:** Yasemin Dinc, Bilge Cetinkaya Demir, Deniz Sigirli, Emel Oguz Akarsu, Furkan Saridas, Bahattin Hakyemez, Mustafa Bakar, Gizem Güllü, Aygül Güneş, Cemile Haki, Emine Rabia Koc, Selcan Akesen, Ezgi Sezer Eryildiz, Özlem Aykac, Zehra Kocabaş Uysal, Atilla Özcan Özdemir, Suat Kamisli

**Affiliations:** a Department of neurology, Uludag University Faculty of Medicine, Bursa, Turkey; b Department of Neurology, Bursa Higher Education and Research Hospital, Bursa, Turkey; c Department of Neurology, Bursa City Hospital, Bursa, Turkey; d Manisa City Hospital, Neurological Intensive Care Clinic, Manisa, Turkey; e Department of Neurology, Eskisehir Osmangazi University Faculty of Medicine, Bursa, Turkey.

**Keywords:** cerebral venous sinus thrombosis, preeclampsia, puerperium

## Abstract

The postpartum period is a well-defined risk factor for cerebral venous sinus thrombosis (CVST). However, it is unclear which patients are at risk for CVST in the postpartum period. Thus, determining some CVST risk factors in postpartum patients may be useful for preventing the disorder in this population. Previous studies have shown that preeclampsia (PE) is a risk factor for pulmonary thromboembolism and deep vein thrombosis, but whether it is related to postpartum CVST has not yet been evaluated. This study aimed to determine if this relationship exists. This study was a case–control study of retrospectively screened patients diagnosed with CVST between 2018 and 2023 at the Uludağ University Faculty of Medicine Department of Neurology, the Eskişehir Osmangazi University Faculty of Medicine and the Bursa City Hospital Health Sciences University Department of Neurology. All of the women who delivered between 2018 and 2023 at the Uludağ University Faculty of Medicine Department of Obstetrics and Gynaecology were included in the control group. In total, 57 out of 322 cases and 4299 out of 4452 controls were included in this study. A nonsignificant relationship was found between CVST and spinal anesthesia, but a significant relationship was found between PE and stillbirth. Women with PE who had recently delivered were found to be at increased risk of developing CVST. The primary limitation of this study is that it was retrospective, and the control group was hospital-based. We recommend that these findings be confirmed by multicenter prospective international studies.

## 1. Introduction

Cerebral venous sinus thrombosis (CVST) is a rare type of thrombosis that occurs due to the occlusion of veins and sinuses draining the brain parenchyma and causing parenchymal damage and hemorrhage. It is mostly seen in young patients, and its incidence rate is 0.2 to 0.5 per 100,000 per year.^[1–10]^ It is a rare cause of venous thromboembolism (VTE), but it is important because it affects young people more frequently and, in some cases, is fatal.^[11]^ The etiology of CVST is multifactorial,^[12]^ and the postpartum period is a well-defined risk factor for CVST.^[13]^ In cohort studies, postpartum patients had been found to constitute 10% to 20% of the entire CVST population.^[14–16]^ However, postpartum CVST is not well understood due to its low incidence. For example, it is unclear which patients are at risk for CVST in the postpartum period. Thus, determining some CVST risk factors in postpartum patients may be useful for preventing the disorder in this population. Previous studies have shown that preeclampsia (PE) is a risk factor for deep vein thrombosis and pulmonary thromboembolism, but its relationship to CVST has not been evaluated.^[17]^ The literature includes many case reports and case series on patients who had undergone spinal anesthesia during delivery and developed CVST afterwards.^[18–20]^ However, case–control studies evaluating the relationship between spinal anesthesia, PE and postpartum CVST are lacking. This study aimed to investigate the relationship between postpartum CVST and PE.

## 2. Materials and methods

This study was a case–control study of retrospectively screened CVST-diagnosed patients who were followed up between 2018 and 2023 at the Uludağ University Faculty of Medicine Department of Neurology, the Eskişehir Osmangazi University Faculty of Medicine and the Bursa City Hospital Health Sciences University Department of Neurology between 2018 and 2023. Uludag University Health Research Ethics Committee approved our study with the decision number 2024-7/2 dated May 8, 2024. Due to the study’s retrospective design, approval of the informed consent form is not required. We performed all methods according to Helsinki Declaration. The inclusion criteria for the case group were as follows: diagnosed with CVST based on magnetic resonance (MR) imaging and contrast-enhanced MR venography at the Uludağ University Faculty of Medicine Hospital, Eskişehir Osmangazi University and Bursa City Hospital; female; 15 to 49 years old; and diagnosed with CVST within 6 weeks after they gave birth. The exclusion criteria for the case group were as follows: male and diagnosed with CVST due to oral contraceptive use, hormone replacement therapy, Behçet’s disease, connective tissue disease, malignancy or other causes. The inclusion criteria for the control group were as follows: had given birth at the Uludağ University Faculty of Medicine Hospital between 2018 and 2023; 15 to 49 years old; and had routine outpatient clinic follow-ups in the obstetrics and gynecology department during their postpartum period. The exclusion criteria for the control group were as follows: had no access to postpartum data of puerperal women and diagnosed with CVST during their pregnancy or postpartum period. The *postpartum period* was defined as the first 6 weeks after a term birth.^[21]^ A *stillbirth* was described as a baby who died before or during delivery after 28 weeks of gestation.^[22]^

A maternal–fetal medicine specialist diagnosed PE according to the American Collage of Obstetric and Gynecology criteria.^[23]^ The type of delivery, the type of anesthesia administered during the delivery and whether the patient had a history of PE were queried from the history of all of the patients in the hospital’s information system. All of the patients were diagnosed with CVST via contrast-enhanced cranial MR imaging and were examined by a neurologist in the emergency department. The patients’ complaints and neurological examinations, whether they had an epileptic seizure and the number of days before they delivered were recorded in their epicrisis. All of them were admitted to the neurology department and evaluated for rheumatologic disease and hereditary hypercoagulopathy. Their clinical outcomes were determined on the third month after their CVST diagnosis based on their modified Rankl score (mRs), with an mRs of 0 to 2 denoting a favorable clinical outcome and an mRs of 3 to 6 denoting an unfavorable clinical outcome. The clinical properties of the postpartum patients with and without CVST were compared, based on which the CVST risk factors were determined.

## 3. Statistical analyses

The statistical analysis was conducted using the IBM SPSS Statistics 28.0 package (IBM Corp., Armonk). The Shapiro–Wilk test was used to determine the normality of the data distribution. The mean ± standard deviation values were calculated for the normally distributed variables, and median (minimum–maximum) values were calculated for the non-normally distributed variables. An independent sample *t*-test was used to analyze the variables that were normally distributed between the 2 independent groups, and the Whitney *U* test was used for the non-normally distributed variables. The Pearson chi-square test or Fisher exact test was used to evaluate the categorical variables and to compare the groups. A relationship was defined as statistically significant when *P* < .05.

## 4. Results

**Figure 1.**
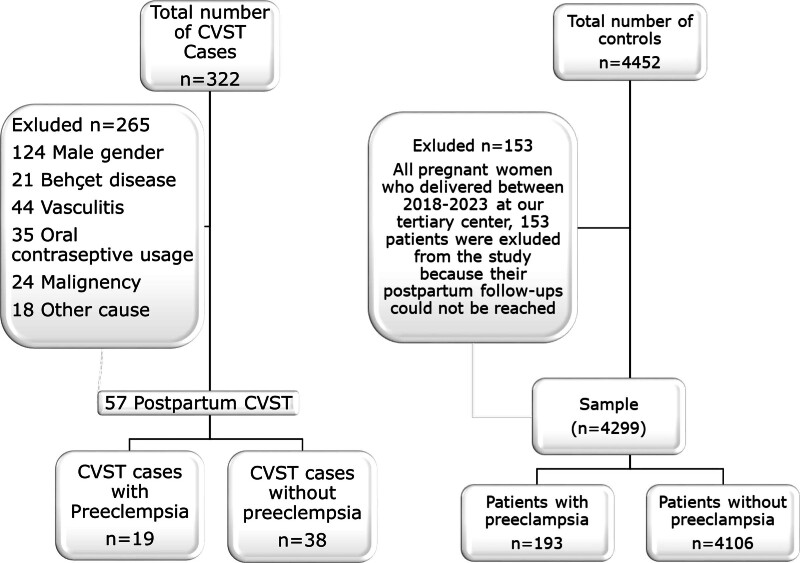
Flowchart shows CVST cases and control group included in the study.

A total of 323 cases were initially available (201 from the Uludag University Faculty of Medicine, 60 from Bursa City Hospital, and 62 from the Eskişehir Osmangazi University Faculty of Medicine), and all 4549 women who gave birth between 2018 and 2023 at the Uludağ University Faculty of Medicine Department of Obstetrics and Gynaecology were included as the controls. The patient data were accessed from the birth statistics in the hospitals’ operating systems. All of the epicrises of the patients in the case and control groups were examined. After the exclusion criteria were applied, 57 cases and 4299 controls remained (Fig. 1). When the risk factors associated with postpartum CVST were evaluated between the control and case group a significant relationship was observed between the presence of stillbirth (*P* < .001) and the presence of preeclampsia (*P* < .001). Meanwhile, no significant relationship was found between age, type of delivery, spinal anesthesia, multiple pregnancy (*P* > .05), (Table 1). When patients with postpartum CVST were examined, 20 (35.08%) patients had vaginal delivery, 28 (49.12%) patients had a history of spinal anesthesia, 8 (14.03%) patients had a stillbirth, 19 (33.33%) patients had PE, and 2 (3.51%) patients had a history of multiple pregnancy. When the initial clinical symptoms of the patients were examined, 19 (33.33%) patients had intracranial hypertension, 39 (68.42%) patients had focal neurological deficits, and 29 (50.87%) patients had epileptic seizures. When the affected vessels were examined, 42 (73.68%) patients had superior sagittal sinus, 36 (63.15%) patients had transverse sinus, 10 (17.54%) patients had sigmoid sinus, 8 (14.03%) patients had sinus rectus, 5 (8.77%) patients had inferior sagittal sinus, and 8 (14.03%) patients had jugular vein thrombosis. There were 42 (73.68%) patients with parenchymal lesions, 15 (26.31%) patients had nonvenous infarction, and 27 (47.36%) patients had hemorrhagic infarction. 36 (63.15%) patients had favorable clinical outcomes. 19 (33.33%) patients had unfavorable clinical outcomes. Death due to CVST occurred in only 1 (1.75%) patient. There was a significant statistical relationship between PE and postpartum CVST. For this reason, postpartum CVST patients with and without preeclampsia were compared. When the clinical, radiological and demographic features associated with postpartum CVST were evaluated between patients with PE or not, a significant relationship was observed between the presence of subarachnoid hemorrhage (*P* = .042). Meanwhile, no significant statistical relationship was found between age, presence of intracranial hypertension, presence of focal syndrome, presence of epileptic seizure, presence of hereditary hyper coagulopathy, spinal anesthesia, involvement of superior sagittal sinus, involvement of transverse sinus, involvement of sigmoid sinus, involvement of sinus rectus, involvement inferior sagittal sinus, involvement jugular vein, thrombosis of cortical vein involvement, number of vessels, presence of parenchymal lesion, presence of nonhemorrhagic venous infarct, presence of hemorrhagic infarction, presence of juxta-cortical hematoma, large parenchymal hemorrhage, clinical outcome (*P* > .05) (Table 2).

**Table 1:** Comparison of patients with and without a history of cerebral venous thrombosis in postpartum patients.

	Postpartum patients with CVST (N = 57)	Postpartum patients without CVST (N = 4299)	*P*-value
Age* mean (min–max)	29.00 (17–41)	30.00 (15–49)	.238
Type of delivery† (vaginal delivery)	20 (35.08%)	1602 (37.26%)	.736
Spinal anesthesia	30 (52.63%)	1810 (42.10%)	.110
Still birth†	8 (14.03%)	91 (2.12%)	**<.001**
Preeclampsia†	19 (33.33%)	193 (4.49%)	**<.001**
Multiple pregnancy†	2 (3.51%)	156 (3.63%)	1.00

Significant differences were shown in bold.

Abbreviations: CVST = cerebral venous sinus thrombosis, ns = nonsignificant, SD = standard deviation.

* Mann–Whitney *U* test.

† Pearson chi-square test/continuity-corrected chi-square test/Fisher exact test.

**Table 2:** Comparison of patients with and without preeclampsia in patients with cerebral venous thrombosis.

	CVST patients with preeclampsia (n = 19)	CVST patients without preeclampsia (n = 38)	*P*-value
Age* (mean ± SD)	30,21 ± 7.33	29.00 ± 4.73	.518
Intracranial hypertension†	8 (42.10%)	11 (28.94%)	.321
Focal syndrome†	11 (57.90%)	28 (73.68%)	.227
Epileptic seizure†	9 (47.36%)	20 (52.63%)	.708
Herediter hypercoagulopathy	10 (52.63%)	21 (55.26%)	.851
Spinal anesthesia†	9 (47.36%)	19 (50.00%)	.851
Superior sagittal sinus†	14 (73.68%)	24 (63.15%)	1.000
Transverse sinus†	13 (68.42%)	23 (60.52%)	.560
Sigmoid sinus†	8 (42.10%)	8 (21.05%)	.095
Sinus rectus†	3 (15.78%)	5 (13.15%)	1.000
Inferior sagittal sinus†	2 (10.52%)	3 (7.89%)	1.000
Jugular vein†	2 (10.52%)	6 (15.78%)	.706
Cortical vein involvement†	12 (63.15%)	21 (55.26%)	.569
Number of vessels* mean (min–max)	2.00 (1–6)	1.97 (1–6)	.667
Parenchymal lesion†	12 (63.15%)	31 (81.57%)	.192
Nonhemorrhagic venous infarct†	6 (31.57%)	9 (23.68%)	.523
Hemorrhagic infarct†	6 (31.57%)	21 (55.26%)	.091
Juxta-cortical hematoma†	3 (15.78%)	11 (28.94%)	.343
Large parenchymal hemorrhage†	3 (15.78%)	10 (26,31%)	.510
Subarachnoid hemorrhage†	0 (0%)	8 (21.05%)	**.042**
Day* mean (min–max)	4.0 (3–21)	9.50 (1–35)	.452
Clinical outcome† (unfavorable)	5 (26.31%)	14 (36.84%)	.427

Significant differences were shown in bold.

Abbreviations: CVST = cerebral venous sinus thrombosis, ns = nonsignificant, SD = standard deviation.

* Mann–Whitney *U* test.

† Pearson chi-square test/continuity-corrected chi-square test/Fisher exact test.

## 5. Discussion

In the postpartum patient population in the current study, we found a nonsignificant relationship between spinal anesthesia and CVST but a statistically significant relationship between stillbirth, PE and CVST. The cesarean section rate in the control group was 62.7%. In a study that evaluated 6,161,976 pregnant women according to Turkey 2018 to 2023 data, the cesarean section rate was 57.55%,^[24]^ and the control group’s cesarean section rate was similar to that of the general population of Turkey. A worldwide systematic review reported PE in 4.6% of all pregnancies^[25]^ and approximately 5% of all pregnancies in the United States.^[26]^ In the current study, the rate of PE in the control group was 4.5%, which is similar to that of the general population. However, the relationship between spinal anesthesia and CVST is unknown, and the former may cause intracranial hypotension, in which dilatation occurs in the venous sinuses as a compensatory measure and slows the flow. According to Virchow triad, slower flow may cause stasis and hypercoagulation.^[27–29]^ Spinal anesthesia has been pointed out as the trigger of CVST in many case reports and case series in the literature,^[18–20]^ but not in any case–control study. The current study showed a nonsignificant relationship between spinal anesthesia and CVST in the postpartum CVST patient group. This may be due to the increased experience of the operator with spinal anesthesia and the fact that spinal anesthesia rarely causes intracranial hypotension.

Although the relationship between PE and VTE has been well evaluated, the relationship of PE with CVST – a cause of VTE – had not been evaluated. Although 20% of the CVST population are postpartum patients, the relationship between CVST and PE had been ignored in most studies. However, in the current study, a statistically significant relationship was found between PE and postpartum CVST. In large cohort studies, it was found that PE increases the risk of VTE fourfold.^[30]^ The possible reason for PE causing VTE is thought to be endothelial dysfunction, the causes of which, in turn, include decreased expression of anticoagulant proteins such as the endothelial protein C receptor, thrombomodulin and the tissue plasminogen activator; impaired anticoagulant activity of activated protein C; and increased expression of adhesion molecules such as the intercellular adhesion molecule-1 (ICAM-1).^[17]^ Through these mechanisms, PE contributes to increased hypercoagulability during pregnancy and the puerperium.

In the current study, another variable that was seen as having a statistically significant relationship to CVST in postpartum patients is a history of stillbirth. In large cohort studies, stillbirth was found to be a risk factor for VTE in the postpartum period.^[31]^ However, CVST had not been evaluated as a risk factor in the literature. Possible reasons for stillbirth being a risk factor for VTE include the patient’s coagulopathy, placental factors and microthrombi.

In population-based studies, it was determined that the risk of VTE increases fivefold during pregnancy and 60-fold during the postpartum period.^[32]^ Silvis et al found that the risk of CVST increases 3-fold during the postpartum period.^[13]^ A previous study on the risk factors for postpartum CVST found that the presence of infection, high maternal age and excessive nausea and vomiting due to pregnancy are risk factors for the development of postpartum CVST.^[33]^ Another study discovered that cesarean delivery creates a predisposition for the development of CVST.^[34]^ In the current study, however, we did not find a significant relationship between postpartum CVST and cesarean delivery. In addition, according to our data, the average ages of mothers with and without CVST had no statistically significant difference. According to the postpartum CVST subgroup analysis of the Action-CVST study, patients with postpartum CVST had more frequent epileptic seizures and parenchymal hematoma and required more frequent neurosurgical procedures.^[35]^ Epileptic seizures were also associated with postpartum CVST in other studies.^[36]^ In another recently published study, the postpartum period was found to be an independent risk factor for cortical vein thrombosis.^[37]^ When PE and non-PE patients were compared among all the postpartum patients, they were found to have a statistically significant similarity, although parenchymal hemorrhages were slightly higher in the non-PE group. There was no difference in their clinical, radiological and demographic characteristics.

## 6. Conclusion

In the current study, we found that spinal anesthesia is not a risk factor for postpartum CVST, whereas PE is. The primary limitation of this study is that it was retrospective, and the control group was hospital-based. We recommend that these findings be confirmed by multicenter prospective international studies.

## Author contributions

**Conceptualization:** Yasemin Dinç, Özlem Aykac.

**Data curation:** Yasemin Dinç, Deniz Sigirli, Selcan Akesen, Ezgi Sezer Eryildiz, Özlem Aykac, Zehra Kocabaş Uysal.

**Formal analysis:** Yasemin Dinç, Deniz Sigirli, Gizem Güllü, Ezgi Sezer Eryildiz, Zehra Kocabaş Uysal.

**Funding acquisition:** Yasemin Dinç.

**Investigation:** Yasemin Dinç, Aygül Güneş, Emine Rabia Koc.

**Methodology:** Yasemin Dinç, Deniz Sigirli, Emel Oguz Akarsu, Mustafa Bakar, Gizem Güllü, Aygül Güneş, Emine Rabia Koc.

**Project administration:** Mustafa Bakar, Gizem Güllü.

**Resources:** Bahattin Hakyemez, Mustafa Bakar, Gizem Güllü, Cemile Haki, Selcan Akesen.

**Software:** Bahattin Hakyemez, Mustafa Bakar, Cemile Haki.

**Supervision:** Bilge Cetinkaya Demir, Bahattin Hakyemez, Mustafa Bakar, Cemile Haki, Atilla Özcan Özdemir, Suat Kamisli.

**Validation:** Bilge Cetinkaya Demir, Furkan Saridas, Bahattin Hakyemez, Mustafa Bakar, Aygül Güneş, Cemile Haki.

**Visualization:** Yasemin Dinç, Bilge Cetinkaya Demir, Furkan Saridas, Bahattin Hakyemez, Aygül Güneş, Cemile Haki, Selcan Akesen.

**Writing – original draft:** Yasemin Dinç, Emel Oguz Akarsu, Furkan Saridas, Bahattin Hakyemez, Cemile Haki.

**Writing – review & editing:** Yasemin Dinç, Emel Oguz Akarsu, Furkan Saridas, Bahattin Hakyemez, Cemile Haki, Emine Rabia Koc, Zehra Kocabaş Uysal, Atilla Özcan Özdemir, Suat Kamisli.
